# Effect of post heat treatment on microstructure and mechanical properties of hot-rolled AA2017 aluminum alloy

**DOI:** 10.1016/j.heliyon.2024.e40922

**Published:** 2024-12-04

**Authors:** Mauro Carta, Leila Aydi, Pasquale Buonadonna, Donato Morea, Mohamad El Mehtedi

**Affiliations:** a Department of Mechanical, Chemical and Material Engineering, University of Cagliari, Via Marengo 2, 09123, Cagliari, Italy; b National School of Engineers of Sfax, University of Sfax, BPW, 3036, Sfax, Tunisia

**Keywords:** AA2017, Quenching, Artificial aging, Microstructure, Mechanical properties

## Abstract

This study investigates the effects of heat treatment, involving solubilization and aging, on the microstructure of AA2017-T451 aluminum alloy. Samples of 4 mm thick rolled plate of AA2017 underwent solution treatment at 500 °C for two different durations, namely 2 h and 6 h, followed by either water quenching (WQ) or air quenching (AQ). Subsequently, they were artificially aged (AA) at 175 °C for 8 h. The samples were investigated in terms of microstructures using techniques such as optical microscopy (OM), scanning electron microscopy (SEM), and energy-dispersive spectroscopy (EDS) and in terms of mechanical properties. Hardness measurements (HV) and tensile tests were conducted on samples oriented along different directions (rolling RD and transverse TD directions). Best results in terms of mechanical properties were obtained with samples solution treated at 500 °C for 6 h, followed by water quenching and an 8-h artificial aging, exhibiting the highest yield and tensile strength. Fracture analysis revealed predominantly ductile behavior in AA2017, characterized by micro-void nucleation, growth, and coalescence. Additionally, the heat treatment response concerning phase constitutions as a function of input composition, temperature, and time was compared with the data obtained by the JMatPro software. The results highlighted the significant influence of quenching rate post-solution heat treatment, aging time, and temperature on the precipitation-hardening process of AA2017, particularly emphasizing the formation of Al_2_Cu (θ-phase) and Mg_2_Si during the aging process.

**Table:** Abbreviations

SSSS	supersaturated solid solution
YS	Proof stress
UTS	Ultimate tensile strength
A%	Elongation at break
RD	Rolling direction
TD	Transverse direction
ST	Solution heat treatment
AQ	Air quenching
WQ	Water quenching
AA	Artificial ageing

## 1 Introduction

AA2017 (Al-Cu-Mg) is a hard aluminum alloy strengthened by heat treatment, it means that the properties of these alloys can be optimized by applying a series of treatments, such as solution treatment, quenching and natural or artificial ageing [[1], [2], [3]]. Because of their higher strength, good weldability and ductility, the AA2xxx have been widely used for aeronautics and aerospace applications [4]. The effects of heat treatments were recently studied on friction stir welded [5] and diffusion bonded samples [6].

Precipitation strengthening is an important hardening method and is used to improve the strength of some aluminum alloys [[7], [8], [9]]. The age-hardening response is mainly controlled by the precipitation behavior and by the morphology of the meta-stable and stable precipitated phases [10].

According to previous studies [11,12], the types of hardening particles in Al-Cu-Mg alloys are influenced by the Cu to Mg weight ratio. Two precipitation sequences are common in AA 2xxx alloys: SSS → GPzones→ θ′′ (thin discs, fully coherent with the matrix) → θ′ (disc shaped and semi-coherent with the matrix) → θ (Al_2_Cu, which is spherical and incoherent at the precipitate-matrix interface) and SSS → GPB zones → S′′ → S′ → S (Al_2_CuMg) [13]. SSS is the supersaturated solid solution obtained from the solution heat treatment. The first stage of precipitation reaction is the formation of clusters (GP zones). The subsequent precipitation sequence and kinetics in Al–Cu–Mg alloys are discussed in detail by other researchers [[14], [15], [16], [17], [18]]. Thus, the θ′′ phase is considered to be the first independent precipitation phase [19], the plate-shaped θ′ phase, of nominal stoichiometry Al_2_Cu, is the most common and effective strengthening precipitate phase in aluminum alloys [20], which provides significant contribution to the yield strength of 2xxx series aluminum alloys. On these bases, in order to obtain improved mechanical properties and microstructure, aluminum alloys are often subjected to different heat treatments [[21], [22], [23], [24]].

Commercially important aluminum alloys contain copper as the main alloying element and phase reactions that occur are those between the aluminum solid solution and the inter-metallic phases Al_2_Cu and Al_2_CuMg. The precipitate morphology, orientation, spacing and degree of coherency with the matrix are all factors that influence the dislocation motion [25]. A lot of work still needs to be done to fully develop the new generation of alloys based on microalloying. For example, Bahl et al. [26] studied Al–Cu alloys micro-alloyed with Mn and Zr to understand the thermal stability and strengthening mechanism of metastable θ′-Al₂Cu precipitates with interfacial segregation after prolonged thermal exposure. Yang et al. [27] also examined the strengthening effect in Al-Cu-Li alloys due to the presence and dimensions of T_1_ precipitates.

Recent studies on 2xxx alloys are focus on their applicability in additive manufacturing technology, particularly laser-based methods like Selective Laser Melting or Direct Metal Laser Sintering, presents challenges for these alloys due to their susceptibility to cracking during rapid heating and cooling cycles. The high thermal conductivity of aluminum combined with the alloy's sensitivity to solidification cracking makes it difficult to print defect-free parts [[28], [29], [30]].

The aim of this study is to determine the influence of solution heat treatment on microstructures, phases, and mechanical properties of the AA2017 alloy in different conditions. The AA2017 aluminum alloy subject of the present study was selected because of its importance in the aerospace industry and the fact that its strength can be increased considerably by precipitation that takes place during solid solution treatment and aging. In particular, the present work investigated the microstructures and mechanical properties (tensile and hardness) in specimens under as received, solution treated (ST), solution heat treated then artificial aged (ST + AA) conditions. To the best of the authors’ knowledge, a comprehensive study on the effects of heat treatment on the mechanical properties of the AA2017 alloy is missing in literature. Using a combination of experimental techniques, including SEM, EDS, and mechanical testing, to corroborate the results, this study offers an in-depth assessment that goes beyond what has been reported in previous literature. The research provides a deeper understanding of how heat treatment variables, such as treatment duration and quenching methods, affect mechanical properties and fracture behavior.

## 2 Materials and methods

In this study, a rolled plate of AA2017-T451 aluminum alloy with a thickness of 4 mm was used. The temper designation T451 indicates that the rolled AA2017 plate was subjected to a solution heat treatment, followed by a stress relief process by stretching to about 1–3% before natural aging [31]. The chemical composition of the studied material is reported in Table 1.

**Table 1:** Chemical composition of AA2017.

Element	Cu	Mg	Mn	Fe	Si	Zn	Ti	Cr	Al
AA2017 (wt.%)	3.91	0.72	0.74	0.52	0.60	0.01	0.01	0.017	bal.

The samples were subjected to solution treatment (ST) at 500 °C for two different soaking times, 2 h, and 6 h, followed by either water quenching (WQ) or air quenching (AQ), and subsequently artificially aged (AA) at 175 °C for 8h in a muffle furnace.

Test specimens were cut from the plate for microstructural examination, microhardness measurement and tensile testing. For this purpose, the specimens were mechanically ground and polished with standard metallurgical methods. The specimens subsequently were electropolished with Barker's reagent by 5 ml HBF_4_ in 200 ml water in 20 V for 90s.

Optical Microscopy (OM), Scanning Electron Microscopy and Energy Dispersive Spectroscopy (SEM-EDS) investigations provided a first overview of microstructural characteristics of samples. Then mechanical properties have been investigated with both Microhardness and tensile tests. Additionally, X-ray diffraction (XRD) analysis was performed using a Siemens D5000 diffractometer to identify the phases present before and after the heat treatments.

Vickers microhardness (HV) was measured on the plane parallel to longitudinal axis (rolling direction) by applying a load of 200 gf for 15 s. More than ten replications were performed for each testing specimen to calculate an average value of the hardness.

**Fig. 1:**
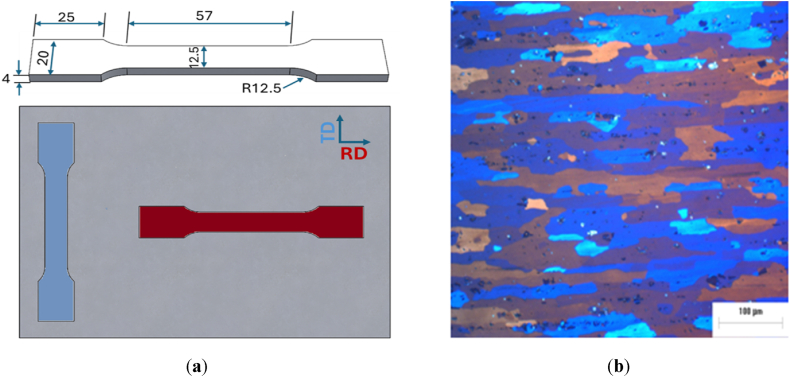
(**a**) Specimen geometry and size (units in mm); (**b**) Optical micrograph of as-received material AA2017 aluminum alloy in the rolling direction.

The tensile specimens were prepared according to ASTM E8/E8M-13a parallel to the rolling direction (RD), and transverse direction (TD), according with the geometry shown in Fig. 1a. The tests were conducted at room temperature and a strain rate of 3 mm/min, and at least three specimens were tested for each condition.

The fracture surface of tensile specimens was examined by Field Emission-Scanning Electron Microscope (FE-SEM).

## 3 Results and discussion

### 3.1 Analysis of time-temperature-transformation and continuous-cooling- transformation curves using JMatPro

Time-temperature-transformation (TTT) curves are beneficial to predicting schedules of solution and age-hardening heat treatments. Continuous cooling transformation (CCT) curves are of interest to determine allowable cooling rates and intervals, either to obtain desired microstructures or to avoid deleterious precipitates in quenching processes following solution treatment of wrought alloys to avoid residual stress or distortion.

**Fig. 2:**
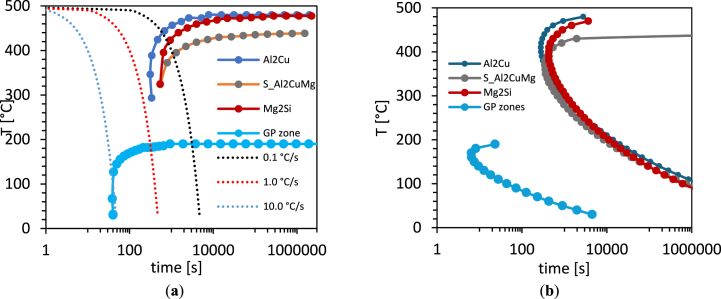
Analysis of precipitates of AA2017 simulated by JMatPro software. (**a**) CCT diagram phases; (**b**) TTT diagram phases.

The CCT curves (with their superposed rates of cooling) clearly describe the relationships of cooling and microstructure (Fig. 2a). The CCT curves reveal the critical cooling rate of the AA2017 for a rapid quenching is around 10 °C/s without phases precipitation. On the other hand, the presence of the precipitation of GP zone for a cooling rate higher of 10 °C/s is noticed. Finally, for a slower quenching rate there is a phase precipitation Al_2_CuMg, Mg_2_Si and Al_2_Cu. Thus, the phase transformation decreases with the increase of cooling rate inversely. It can be concluded that the rapid quenching (water quenching) improves the mechanical and microstructure properties of aluminum alloy AA2017 listed in Table 3.

The composition of the as-received AA2017 is presented in Table 1, the AA2017 was analyzed by JMatPro and the TTT diagram is shown in (Fig. 2b). The diagrams display the start of transformation (0.1 % amount of phase formed) of various phases from the supersaturated Al phase. Based on the experimental results only θ-Al_2_Cu and Mg_2_Si were found precipitated. At temperatures below 180 °C, the GP zone formation is favored, and the kinetics is very rapid. In Fig. 2a of the AA2017 CCT diagram, two different cooling rates of 0.1 °C/s and 1 °C/s are presented. At a cooling rate of 1 °C/s, no phases precipitate, indicating that this is the minimum cooling rate required to obtain a supersaturated solid solution (SSSS). Conversely, at a cooling rate of 0.1 °C/s, all phases precipitate.

### 3.2 Evolution of microstructure during heat treatment

The evolution of the microstructure during heat treatment was analyzed by OM and SEM-EDS. Fig. 1b Shows a typical microstructure of AA2017 at the T451 condition in the rolling direction (RD). Microstructure consists of α-Al matrix with elongated grains in the rolling direction and small secondary particles that are formed due to natural aging of the alloy (T451 condition.

**Fig. 3:**
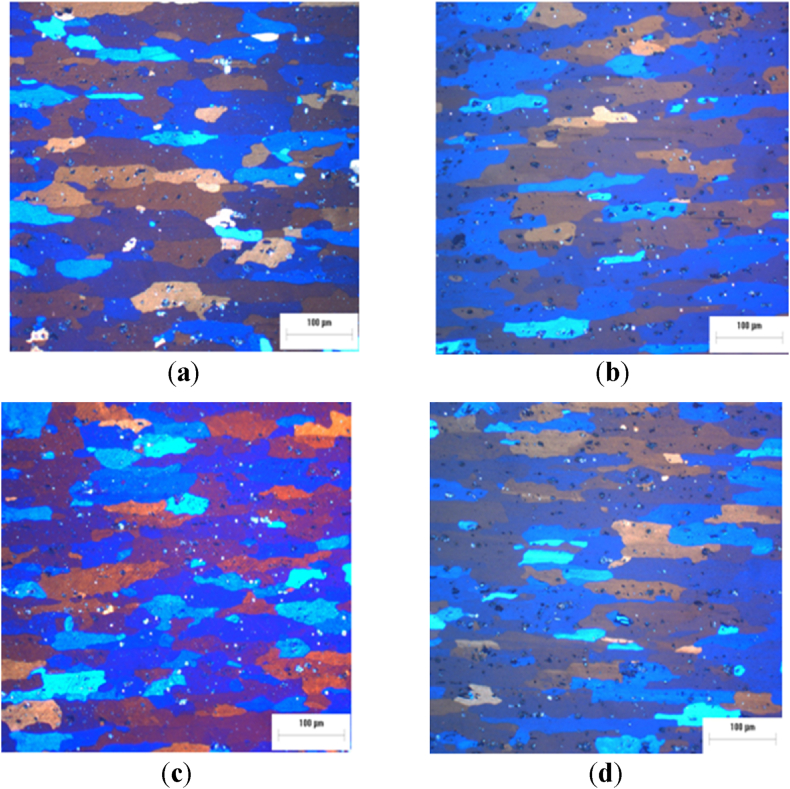
Optical micrograph of AA2017 aluminum alloy ST at 500 °C for 2h (**a**) AQ, (**b**) WQ, (**c**) AQ then AA at 175 °C for 8h (**d**) WQ then AA at 175 °C for 8h.

Fig. 3a-d shows the microstructure following solution treatment (ST) at 500 °C for 2 h, succeeded by water quenching (WQ) or air quenching (AQ), and artificial aging (AA.

**Fig. 4:**
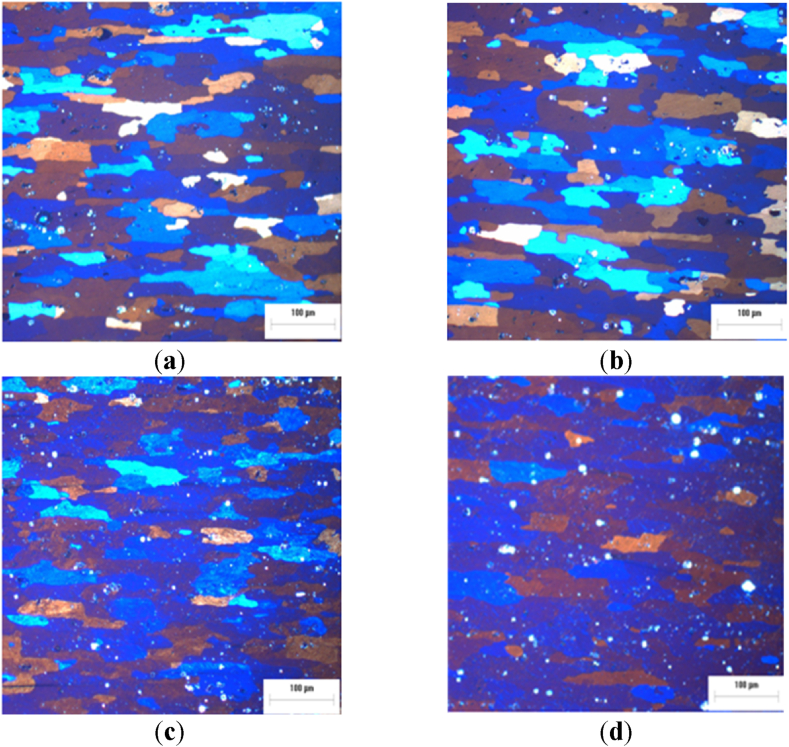
Optical micrograph of AA2017 aluminum alloy ST at 500 °C for 6h (**a**) AQ, (**b**) WQ, (**c**) AQ then AA at 175 °C for 8h, (**d**) WQ then AA at 175 °C for 8h.

Fig. 4a-d the solution treatment time is extended to 6 h, followed by WQ or by AQ, then AA treatment. Moreover, after ST at 500 °C for 6h. Furthermore, although some grain becomes equiaxed due to static recrystallization phenomenon, in the specimen at 500 °C for 6h, the elongated grains are retained in the specimen at 500 °C for 2h.

All images in Fig. 4 exemplify this recrystallization, showing the growth of new equiaxed grains while still retaining the texture imparted by rolling. Additionally, these figures reveal a high density of bright particles that are important to the alloy's strength by impeding dislocation movement, which will be discussed in detail in the subsequent sections, thanks to the EDS analysis.

EDS analysis conducted in a previous study on an AA2017 revealed the presence of iron-rich precipitates like Al_3_Fe or Al_12_(Fe,Mn)_3_Si, along with Al_2_Cu(θ) and Al_2_CuMg(S) phases [11]. These precipitates are expected to form according to the JMatPro curves (Fig. 2), depending on the cooling rate.

EDS analysis and mapping were conducted to identify the formation of intermetallic compounds. EDS analysis of the observed precipitates helped identify them, with atomic percentages for Al₂Cu, for example, showing an approximate 2:1 ratio of aluminum to copper. EDS mapping further confirmed this by demonstrating that these elements were co-located within the precipitates. Additionally, the morphologies of the precipitates support this identification, as they closely match those reported in the literature for Al₂Cu formations [11,32].

**Fig. 5:**
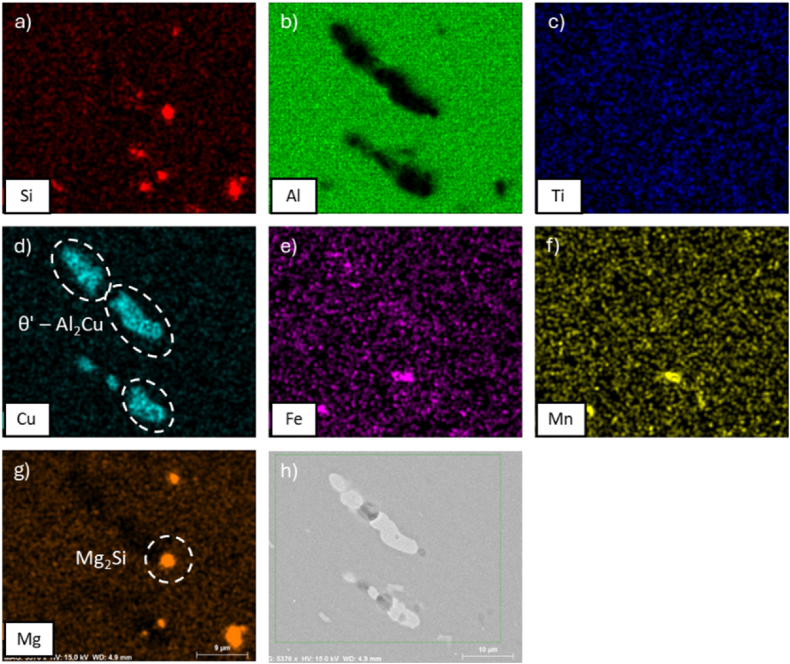
EDS maps of alloy AA2017 after ST at 500 °C for 2h followed by WQ, showing element distributions: (a) Si, (b) Al, (c) Ti, (d) Cu, (e) Fe, (f) Mn, (g) Mg, and (h) SEM analyzed region.

**Fig. 6:**
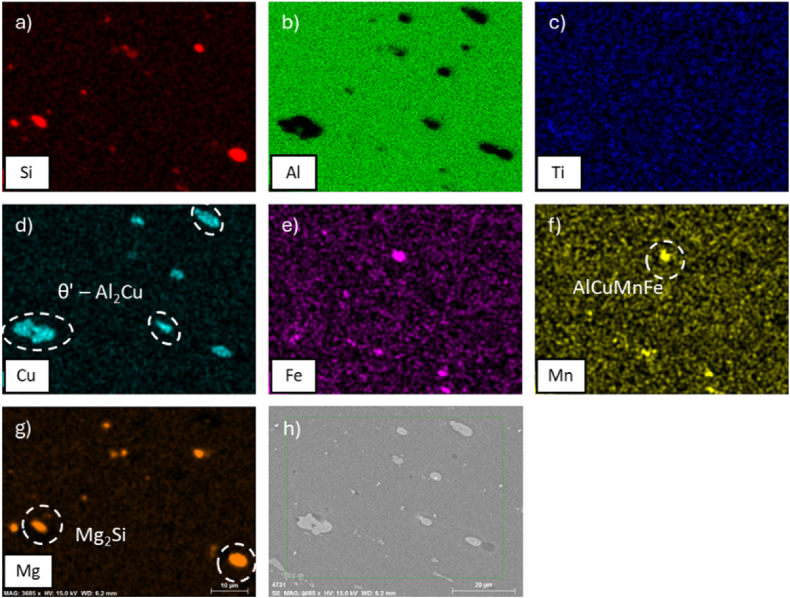
EDS maps of alloy AA2017 after ST at 500 °C for 2h, WQ then AA at 175 °C for 8h**.** In particular: (a) Si, (b) Al, (c) Ti, (d) Cu, (e) Fe, (f) Mn, (g) Mg, and (h) SEM analyzed region.

Comparable outcomes have been observed in the present study, which additionally identifies the presence of Mg_2_Si precipitates. This is attributable to the relatively elevated concentrations of Si and Mg in the alloy composition (Table 1). As shown in Fig. 5 (sample after ST at 500 °C for 2h then WQ), and Fig. 6 (after ST followed by WQ then AA) the precipitates that only light up in the Cu map are almost certain to be of θ′-Al_2_Cu type, as the θ-Al_2_Cu phase would be incoherent and very large. This has been reported before in pure Al-Cu alloys [33]. It can be observed the presence of inter-metallic phases (AlCuMnFe) and Mg_2_Si. No S phase (S-Al_2_CuMg) was observed.

**Fig. 7:**
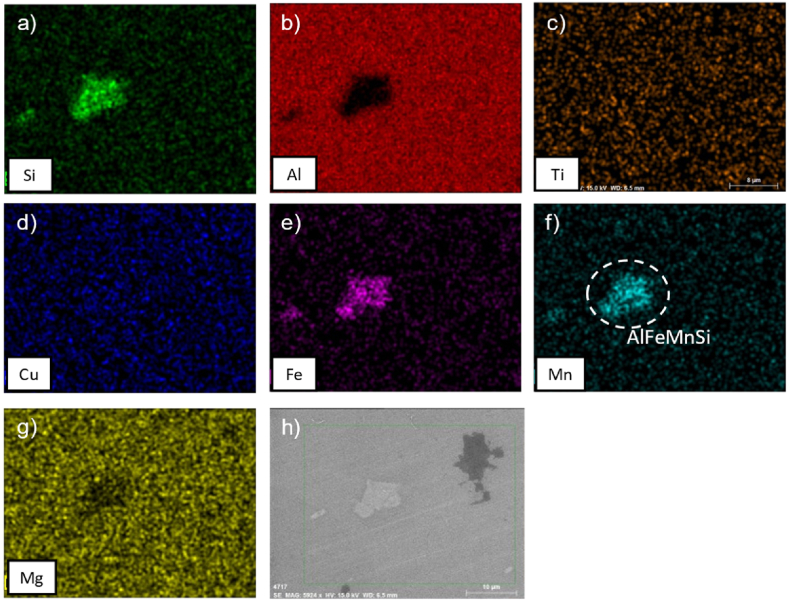
EDS maps of alloy AA2017 after ST at 500 °C for 6h followed by WQ, showing element distributions: (a) Si, (b) Al, (c) Ti, (d) Cu, (e) Fe, (f) Mn, (g) Mg, and (h) SEM analyzed region.

**Fig. 8:**
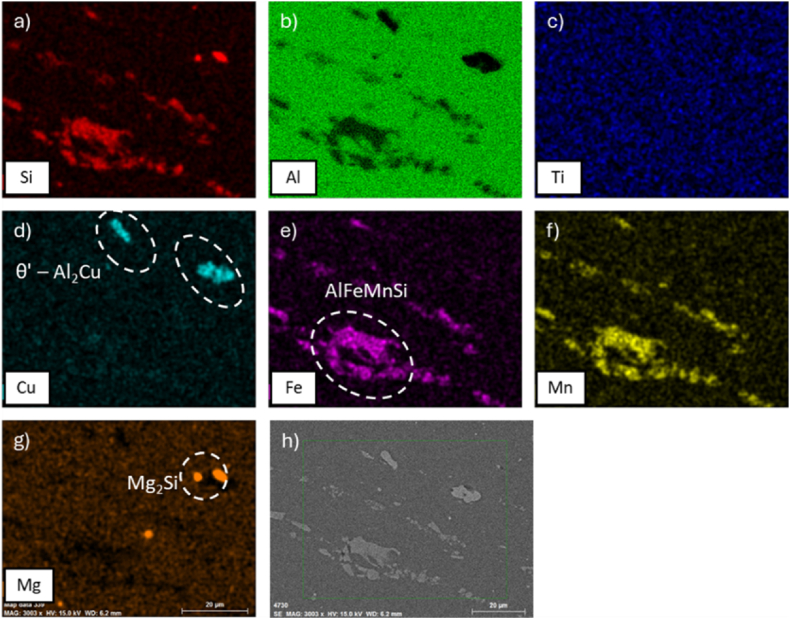
EDS maps of alloy AA2017 after ST at 500 °C for 6h, WQ then AA at 175 °C for 8h. In particular, element distributions: (a) Si, (b) Al, (c) Ti, (d) Cu, (e) Fe, (f) Mn, (g) Mg, and (h) SEM analyzed region.

It can be observed from Fig. 7, Fig. 8 that the samples after ST at 500 °C for 6h then WQ and after ST at 500 °C followed by WQ then AA, show the presence of iron-rich precipitates such as Al_3_Fe or Al_12_(Fe,Mn)_3_Si, which is in agreement with [11]. It is noted that iron-rich compounds are almost insoluble; in fact, they remain visible even after the solution treatment for 6 h. However, after 6 h of solution treatment and WQ, the other intermetallics are not visible, suggesting the formation of a supersaturated solid solution (SSSS) [[34], [35], [36]].

As already mention, the α-Al phase refers to the solid solution of copper and other alloying elements in the FCC lattice of aluminum, while the θ phase refers to the intermetallic compound conforming to the chemical formula Al_2_Cu [34,[37], [38], [39]].

During the aging process, both θ(Al_2_Cu) and S(Al_2_CuMg) phases should precipitate out of the solid solution and strengthen the alloy. The fractions of these two phases depend on the concentration ratio of Cu to Mg, as well as their total content according to Refs. [24,40]. Similarly to the 2 h heat treatment, no S phase was observed under this condition.

The implications for the mechanical properties are significant and will be discussed in the following.

**Fig. 9:**
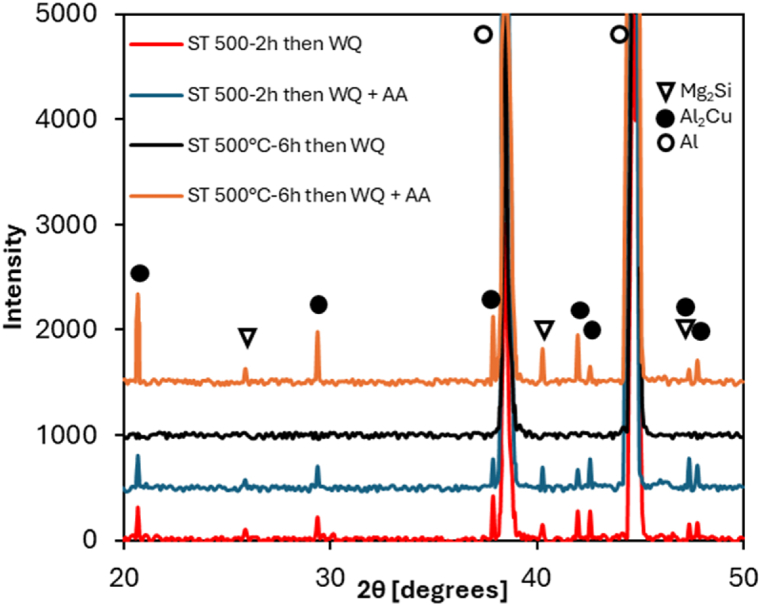
XRD patterns under different HT conditions.

The X-ray diffraction (XRD) patterns presented in Fig. 9 show the phase evolution of AA2017 aluminum alloy under different heat treatment conditions. The results complement the EDS analysis. The red curve, representing solution treatment (ST) at 500 °C for 2 h followed by water quenching (WQ), shows relatively low intensity of precipitate peaks, with the Al peaks being the most dominant. This indicates that the solution treatment is insufficient to dissolve all the precipitates, leaving the alloy with Cu and Mg still present in precipitate form. The black curve, representing solution treatment (ST) at 500 °C for 6 h followed by water quenching (WQ), shows no precipitate peaks, with the Al peaks remaining dominant. This suggests that, after quenching, the alloy is largely in a supersaturated solid solution state, with most of the solute atoms (such as Cu and Mg) dissolved in the aluminum matrix rather than precipitated.

The blue and orange curves, representing samples subjected to both solution treatment and artificial aging (AA), show significant differences compared to the quenched-only samples. In the samples treated for 2 h plus AA, the aging process results in the coarsening of the precipitates that were not fully dissolved after the 2-h solution treatment. The artificial aging leads to a significant increase in the intensity of peaks corresponding to Al₂Cu and Mg₂Si phases, especially in the orange curve, where the alloy underwent solution treatment for 6 h followed by AA. This extended treatment enhances the precipitation of strengthening phases, leading to improved mechanical properties of the alloy, as described in the subsequent analysis.

### 3.3 Evolution of mechanical properties during heat treatment

#### 3.3.1 Microhardness results

**Fig. 10:**
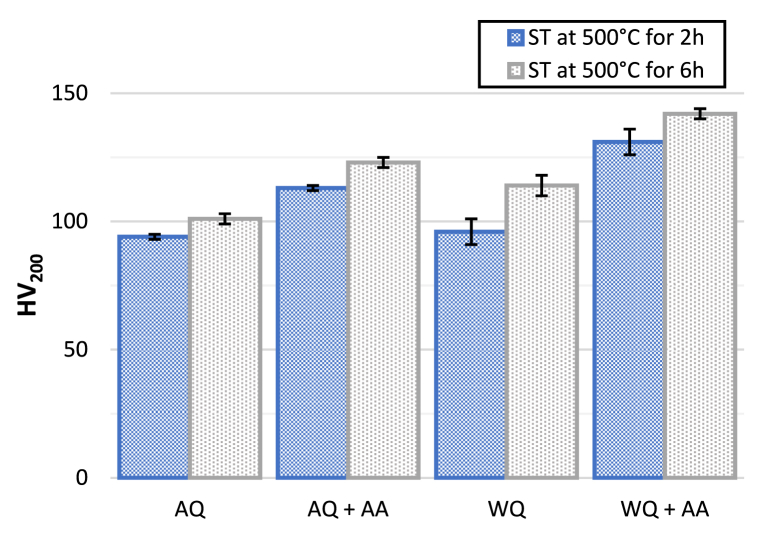
Measurement of hardness for the AA2017 in all the studied conditions.

Table 2 and Fig. 10 show the change in hardness for all the conditions studied compared to the as-received material. From the hardness measurements reported in Table 2, the value of as-received AA2017 was measured to be 128 HV. It can be noted that such a high value indicates the hardening process that occurs during natural aging and the strengthening effect due to the plastic deformation of 1–3% typical of the T451 condition. Therefore, the natural ageing in Al–Cu alloys is known to lead to a significant hardening [41].

**Table 2:** The microhardness (HV) of AA2017 for all samples.

AA2017		Hardness (HV200)
T451	As-received	128 ± 4

ST at 500 °C for 2h	AQ	94 ± 1
	AQ + AA	113 ± 1
	WQ	96 ± 5
	WQ + AA	131 ± 5
ST at 500 °C for 6h	AQ	101 ± 2
	AQ + AA	123 ± 2
	WQ	114 ± 4
	WQ + AA	142 ± 2

After ST at 500 °C for 2h then AQ or WQ, the hardness is measured to be 94 HV and 96 HV, respectively, which suggest the partially dissolution of precipitates that initially contributed to hardening in the as-received state. Similarly, after ST at 500 °C for 6 h followed by AQ and WQ, the hardness values are 101 HV and 114 HV, respectively. The higher hardness in WQ samples compared to AQ samples can be attributed to the more rapid quenching rate associated with water quenching, which leads to a higher density of vacancies and a greater degree of supersaturation in the alloy matrix. This supersaturation favors the formation of fine, well-distributed precipitates that obstruct dislocation motion more effectively, resulting in increased hardness after ageing. The lower hardness in AQ samples is due to slower cooling rates, resulting in less supersaturation and coarser precipitates after ageing treatment, which are less effective at hindering dislocation movement and, therefore, contribute less to the overall hardness.

The values of hardness, as expected, increased after AA in all conditions. After ST at 500 °C for 2h, increased respectively to 113 HV and 131 HV for AQ and WQ samples. On the other hand, the values after ST at 500 °C for 6h, increased to 123 HV and 142 HV for AQ and WQ samples, respectively. This increase in hardness with increasing solution time (6 h) can be attributed to a satisfactory degree of solution of the undissolved or precipitated soluble phase constituents, leading to a good homogeneity of solid solution. Others study reported that T6 treated alloys showed a peak hardness of 141 HV after ageing for 9 h at 180 °C, which decreased slightly with further ageing. They have shown that ageing at 180 °C results in optimum age hardening response for T6 temper in Al-Cu based alloys [42,43]. In the present study, a peak hardness of 142 HV was obtained after 8 h of ageing at 175 °C.

As mentioned, after artificial ageing the hardness of water-quenched samples is slightly higher than that of the air-cooled sample. It is known that the speed of quenching has an impact on the mechanical properties of aluminum alloy. The improvement in hardness values after a solution heat treatment and quenching followed by artificial ageing can be attributed to the presence of θ-Al_2_Cu and Mg_2_Si phases, as the degree of irregularity in the lattice leads to an increase in the mechanical properties of the alloy [44].

Compared to WQ, AQ reduced the strength and hardness, this is well reported in literature [45]. This effect is ascribed to the low super saturation resulting from the loss of vacancies during slow quenching and to the precipitation of solutes whose size and distribution can no longer contribute to strengthening [46,47].

#### 3.3.2 Tensile test results

All the mechanical properties, including ultimate tensile strength (UTS), proof stress (YS) and elongation at break (A%), obtained from tensile tests are shown in Table 3 for every condition, for both RD and TD. The first observation from the mechanical properties is that a certain degree of anisotropy consistently occurs even after heat treatments, as indicated by the TD always displaying lower strength than the RD, although not as pronounced as in the as-received (T451) condition. However, in terms of ductility, the results are nearly identical. As the highest hardness conditions are achieved through water quenching, the same is true for mechanical properties where the optimal results are obtained with this type of quenching. Therefore, the discussion will focus on tensile test results from samples subjected to this method of treatment.

**Fig. 11:**
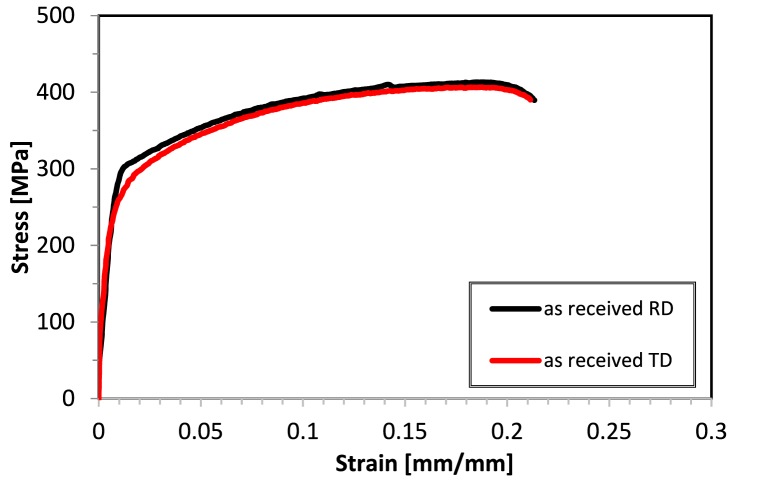
Tensile curves of AA2017 T451 in as-received condition in RD and TD.

**Fig. 12:**
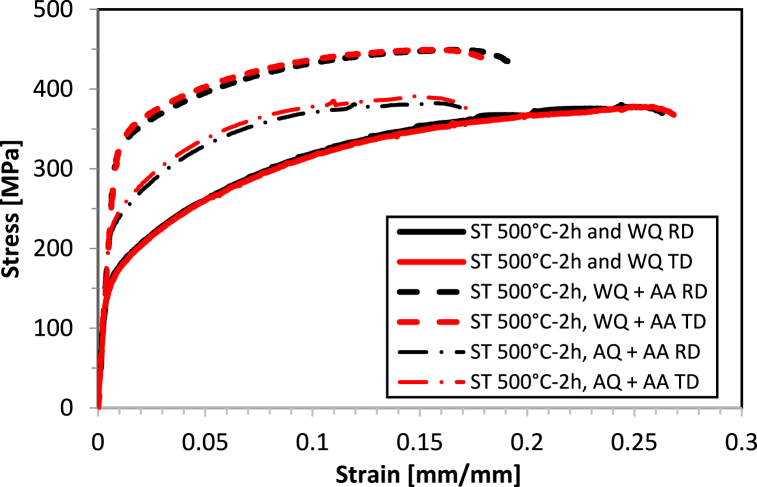
Tensile curves of AA2017 in RD and TD after ST 500 for 2h then WQ and WQ then AA and AQ then AA.

**Fig. 13:**
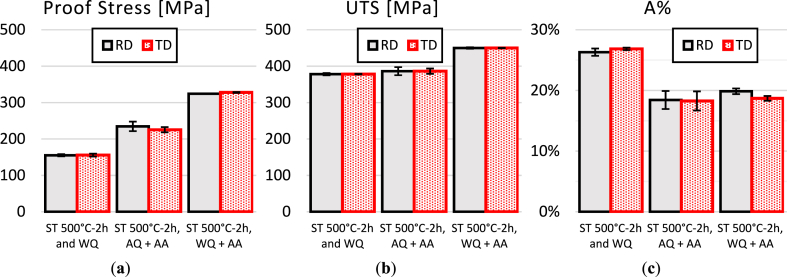
Tensile properties of AA2017 after ST at 500 °C for 2h in RD and TD: (a)Proof stress, (b) UTS and (c) elongation at break.

The stress-strain behavior of AA2017-T451 in two directions, RD and TD, is illustrated in Fig. 11. The as-received material exhibits a proof stress of 290 MPa in RD and 251 MPa in TD, with an Ultimate Tensile Strength (UTS) of 411 MPa in RD and 407 MPa in TD. The elongation percentage (A%) is almost identical in both directions, with 21.0 % in RD and 21.1 % in TD. These properties result from the T451 condition, which is a combination of natural aging and the strengthening effect due to the 2–3% strain applied. These balanced properties suggest good isotropy in tensile behavior in the T451 condition, with the largest difference noted in proof stress but almost identical behavior in terms of elongation (A%) and ultimate tensile strength (UTS). Fig. 12 depict the strength behavior after subjecting the sample to solution treatment at 500 °C for 2 h, followed by water quenching and aging at 175 °C for 8 h or by air quenching and aged under the same conditions. Fig. 13(a–c) illustrate the comparison of mechanical properties of the tested conditions, including proof stress, UTS, and A%, respectively. After solution treatment at 500 °C for 2 h followed by water quenching, there's a significant reduction in proof stress from the as received condition to 155 MPa in RD and 156 MPa in TD, indicating a softening of the material due to heat treatment. The UTS also decreases to 380 MPa in RD and 378 MPa in TD. However, there's an increase in elongation percentage to 26.3 % in RD and 26.8 % in TD, suggesting that the material has become more ductile. This condition demonstrates higher ductility than the T451 condition but lower strength. Solution treatment dissolves the precipitates, and while quenching retains a supersaturated solid solution, the strengthening effect from the T451 condition is lost due to the heat treatment, resulting in lower UTS and proof stress. Sample in ST 500°C-2h, AQ + AA condition shows increased proof stress (235 MPa in RD and 225 MPa in TD) and slightly increased UTS (390 MPa in RD and 386 MPa in TD) compared to WQ but not as high as the as-received material. Elongation percentages are lower (18.4 % in RD and 18.3 % in TD), reflecting a decrease in ductility due to artificial aging. However, sample in ST 500°C-2h, WQ + AA condition exhibits the highest proof stress (324 MPa in RD and 328 MPa in TD) and UTS (451 MPa in RD and 450 MPa in TD) for the 2-h solution treatment, this condition also shows good ductility, with elongation percentages of 19.9 % in RD and 18.7 % in TD. The artificial aging enhance strength significantly while maintaining reasonable ductility. Based on these results, it is evident that air quenching with AA does not significantly improve the mechanical properties, whereas water quenching followed by artificial ageing is markedly superior.

Table 3 reports that the tensile strength of AA2017 in both RD and TD directions is higher after water quenching followed by artificial aging (AA) compared to only WQ. As already known, this suggests that AA has an impact on the tensile properties of AA2017.

**Table 3:** The tensile properties of AA2017 for two directions, RD, and TD.

AA2017	Proof Stress [MPa]		UTS [MPa]		A%	
	RD	TD	RD	TD	RD	TD
As received (T451)	290 ± 2	251 ± 3	411 ± 2	407 ± 2	21.0 ± 0.4	21.1 ± 0.4
ST 500°C-2h and WQ	155 ± 3	156 ± 4	380 ± 3	378 ± 1	26.3 ± 0.6	26.8 ± 0.2
ST 500°C-2h, AQ + AA	235 ± 13	225 ± 7	390 ± 11	386 ± 7	18.4 ± 1.5	18.3 ± 1.6
ST 500°C-2h, WQ + AA	324 ± 1	328 ± 1	451 ± 2	450 ± 1	19.9 ± 0.5	18.7 ± 0.4

ST 500°C-6h and WQ	178 ± 2	161 ± 1	397 ± 3	380 ± 2	29.0 ± 0.7	28.0 ± 0.5
ST 500°C-6h, AQ + AA	189 ± 6	189 ± 1	365 ± 1	372 ± 3	18.6 ± 1.2	18.4 ± 1.5
ST 500°C-6h, WQ + AA	347 ± 2	332 ± 3	454 ± 2	449 ± 2	17.4 ± 0.1	18.1 ± 0.5

**Fig. 14:**
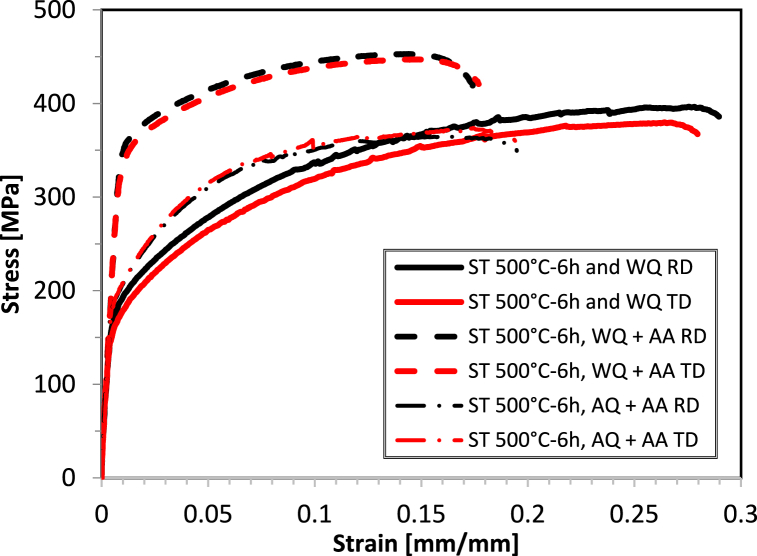
Tensile curves of AA2017 after ST 500 for 6h then WQ and WQ then AA and AQ then AA.

**Fig. 15:**
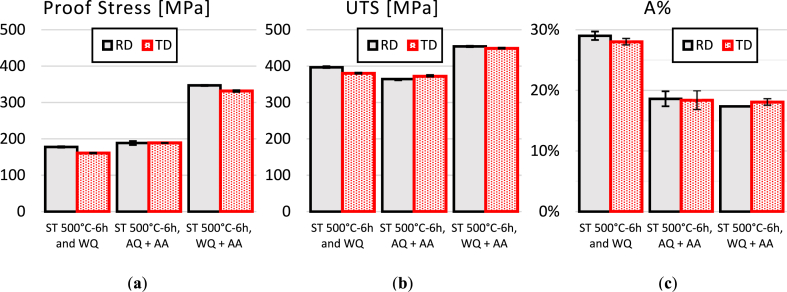
Tensile properties of AA2017 after ST at 500 °C for 6h in RD and TD: (a) Proof stress, (b) Ultimate stress and, (c) elongation at break (A%).

Fig. 14 shows the tensile curves and the mechanical properties of all the studied conditions after 6 h ST. Fig. 15(a–c) present bar charts depicting the tensile properties of AA2017 derived from the curves in Fig. 14. The charts illustrate (a) proof stress, (b) UTS, and (c) A%, respectively. Comparing the as-received condition to the 6h WQ, similar to the 2h WQ, higher ductility was found than in the T451 condition but with lower strength. The solution treatment dissolves the precipitates, and while quenching retains a supersaturated solid solution, the strengthening effect from the T451 condition is lost due to the heat treatment, resulting in lower\ UTS and proof stress. Solution Treatment for 6 Hours and Water Quenched (ST 500°C-6h and WQ) leads to a higher proof stress (178 MPa in RD and 161 MPa in TD) and a slight increase in UTS (397 MPa in RD and 380 MPa in TD) compared to the 2-h treatment but improves elongation to 29.0 % in RD and 28.0 % in TD, indicating enhanced ductility. These results show improvements in terms of proof stress, UTS, and A% compared to the 2-h solution treatment (ST) and water quenching (WQ). This result may be due to the higher level of supersaturation obtained after 6 h: the more Cu and Mg atoms remain dissolved in the aluminum matrix the higher concentration of solute atoms contributes to solid solution strengthening, as solute atoms distort the lattice and hinder dislocation movement.

The solution treated samples for 6 Hours, Water Quenched, and Artificially Aged (ST 500°C-6h, WQ + AA) shows the highest proof stress (347 MPa in RD and 332 MPa in TD) and UTS (454 MPa in RD and 449 MPa in TD) among all the tested samples, indicating that the combination of a longer solution treatment time and artificial aging significantly strengthens the material. However, there is a reduction in elongation percentage (17.4 % in RD and 18.1 % in TD), suggesting a trade-off between strength and ductility. Comparing these results with the ST 500°C-6h, AQ + AA condition there's a notably decrease in proof stress (189 MPa in RD and 189 MPa in TD) and UTS (365 MPa in RD and 372 MPa in TD) but a slightly increase in A%. These properties are even lower than in the 2h ST and AQ + AA.

According to hardness measurements, increasing the time of solution treatment to 6h at 500 °C and subsequent aging at 175 °C for 8 h results in an evident improvement in tensile strength for the AA2017 alloy. The ultimate tensile strength values for RD and TD directions reach their maximum at 454 ± 2 MPa and 449 ± 2 MPa, respectively, while yield strength values are 347 ± 2 MPa and 332 ± 3 MPa, respectively. This may be due to the fine distribution of secondary precipitates in the aluminum alloys rich solid solution matrix. The precipitation of Cu is the main responsible for the improvement in properties when applying the treatment of artificial aging. Hence, the difference of the strengths and elongation of both quenching and aging along directions RD and TD is minor, thus indicating slight anisotropy of AA2017 for different heat treatments.

Comparing Hardness and Strength, the sample treated for 6 h shows higher hardness (142 HV) and tensile strength (454/449 MPa UTS) compared to the 2-h treated sample (131 HV hardness and 451/450 MPa UTS). This suggests that the material becomes harder and stronger with a longer solution treatment, which is consistent across both the hardness and tensile test results. However, there is a slight trade-off between hardness and elongation. The 6-h treatment sample, while being harder and stronger, shows a decrease in elongation in the RD (from 19.9 % to 17.4 %), indicating reduced ductility. Similarly, the TD elongation slightly decreases (from 18.7 % to 18.1 %). There is a positive correlation between proof stress and hardness. As the hardness increases with longer solution treatment times, so does the proof stress, which goes from 324 to 328 MPa to 347 and 332 MPa in RD and TD, respectively.

Comparing the Proof Stress of the 6-h solution treated sample to the 2-h treated sample, it exhibits a higher proof stress in both directions, RD and TD compared, indicating an increase in yield strength with longer solution treatment time. However, in terms of UTS there is a slight increase in UTS for the 6-h treated sample in both RD and TD, suggesting that the longer treatment duration may contribute to a marginal improvement in the peak strength. On the other hand, the elongation decreases slightly in the RD for the 6-h treatment compared to the 2-h treatment but increases in the TD. This might suggest that longer solution treatments slightly reduce ductility in the RD while maintaining or slightly improving it in the TD. From these comparisons, it can conclude that extending the solution treatment time from 2 h to 6 h significantly increased the proof stress and marginally the ultimate tensile strength, with a slight decrease in ductility.

The increased hardness in water-quenched samples following artificial aging can be primarily attributed to the rapid quenching process, which prevents the diffusion and coarsening of precipitates. Rapid cooling from solution treatment temperatures facilitates the retention of a high vacancy concentration within the matrix, essential for the subsequent artificial aging process. This results in a highly supersaturated solid solution (SSSS), conducive to the precipitation of finely dispersed θ(Al_2_Cu) and Mg_2_Si phases upon aging. These precipitates, being finely dispersed and coherent with the matrix, effectively obstruct dislocation motion, thereby reinforcing the alloy's mechanical strength. The reduced strength and hardness observed in air-quenched samples corroborate the well-established notion that slower cooling rates diminish supersaturation levels. The gradual reduction in vacancies during slower cooling permits the growth of precipitates to sizes less effective at blocking dislocations. Consequently, the precipitate distribution in AQ samples is inadequate to enhance mechanical properties substantially, as opposed to the WQ samples.

Iron-rich intermetallics, such as those found in the present study containing Fe, Mn, and Si, exhibit a high degree of thermal stability and insolubility. Even after 6 h of solution treatment, these phases persist within the matrix, as evidenced in Fig. 7, Fig. 8. These precipitates, particularly the Al_2_Cu phase, are confirmed to be products of T6 heat treatment and are fundamental to the alloy's precipitation strengthening.

#### 3.3.3 Analysis of fracture behavior of AA2017

**Fig. 16:**
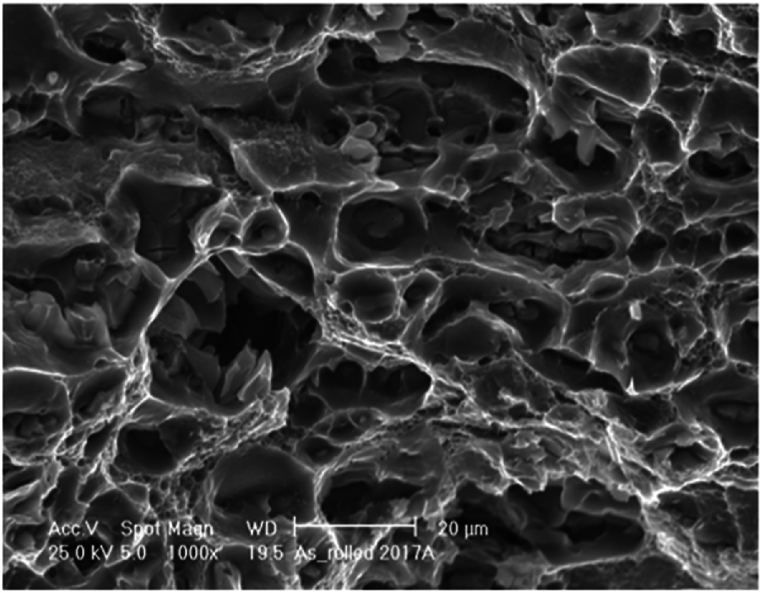
Fracture surface of AA2017 as-received 1000 × magnification.

After the tensile tests were performed, the fracture end of the sample part along RD was cut and carefully observed through SEM to determine fracture morphology. This section will focus on the fracture surfaces of the as-received sample and those that were water-quenched and artificially aged for 2 and 6 h, which are the best conditions obtained. Fig. 16 shows the fracture morphologies of broken sample from tensile tests of AA2017-T451 as-received, along the rolling direction RD. It can be observed that the fracture mode of all samples was ductile fracture, as demonstrated by the large number of small dimples shown on the fracture surfaces. Furthermore, the fracture surfaces of the tensile test specimens evinced deep cavities.

**Fig. 17:**
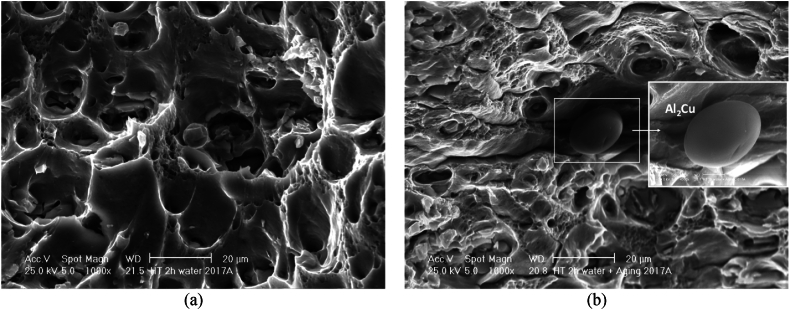
Fracture surface of AA2017 at a ST of 500 °C for 2h (**a**) WQ, (**b**) WQ then AA 1000 × magnification.

Fig. 17a presents the SEM fractography of sample subjected to ST of 500 °C for 2h. At low time (2h) many secondary phases which pre-existed in the provided material, were not completely dissolved into the matrix. Some precipitates were formed and grown from the matrix during ageing (Fig. 17b). The SEM image (Fig. 17a) imply that the dimples in water quenched sample are larger and deeper than the dimples in artificial aged sample (Fig. 17b). The dimples in the water-quenched sample are notably deeper than those in the artificially aged sample. The heat-treated sample (2h ST, WQ, and AA) shows an increase in hardness which correlates with the change in the fracture surface. The fracture surface of this sample indicates a more brittle nature with the presence of precipitates and a mix of ductile and brittle features. This suggests that the heat treatment has increased the material's strength but also has introduced some brittleness as compared to the as-received condition, which is a common trade-off in materials engineering. Despite the increased brittleness suggested by the fracture surfaces, the heat-treated samples still demonstrate considerable ductility in the tensile tests, albeit slightly reduced compared to the as-received material.

**Fig. 18:**
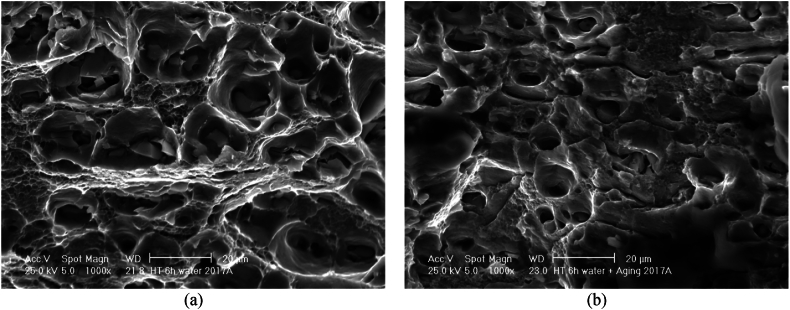
Fracture surface of AA2017 at a ST of 500 °C for 6h (**a**) WQ, (**b**) WQ then AA 1000 × magnification.

Fig. 18a illustrate the fracture morphologies of sample at an ST of 500 °C for 6h then WQ and Fig. 18b after AA at 175 °C for 8h Fig. 18b. These samples were subjected to longer time (6h) of ST compared with sample reported in Fig. 17a and b. Thus, the prolonged period provided enough time for dissolution of the secondary phase, diffusion of atoms and growth of undissolved particles during ageing fine precipitates dissolved from SSSS were uniformly distributed and thus formed shallow dimples, resulting in a slight enhanced strength.

Samples subjected to ST at 500 °C for 2 or 6h, then WQ (Fig. 17, Fig. 18a) with hardness of 96HV and 114HV, respectively, and higher elongation are 26.3 % and 29.0 %, respectively in RD, showed a large and relatively uniform network of bigger and deeper dimples (characteristic of ductile fracture) with round morphology [48]. The as-received material (Fig. 16) exhibited a purely ductile fracture with a rough texture and numerous dimples. Both the 2-h and 6-h treated materials show a similar ductile fracture mode, but with indications that the treatment has affected the microstructure, in fact it can be observed that the size and depth of dimples after ST for 2h then WQ is slightly bigger compared with sample after ST for 6h then WQ.

The age-hardened samples, after ST at 500 °C for 2 or 6 h followed by WQ and then AA, exhibit higher hardness values of 131 HV and 142 HV, respectively, along with relatively low elongation percentages of 19.9 ± 0.5 % and 17.4 ± 0.1 % in the RD, respectively. The ductility in the TD is almost unchanged, at 18.7 ± 0.4 % and 18.1 ± 0.5 %, respectively. They exhibited ductile tearing with few discernible cleavage planes (Fig. 17, Fig. 18b). The fracture surfaces displayed a mixed structure composed of irregular dimples with particles observed at the bottom and parabolic profiles, which contribute to the combination of strength and ductility [49,50]. Furthermore, the size and depth of the fracture dimple is related to the hardness of the material, and the plasticity behavior of metal changes with temperature [51]. The formation of these dimples is due to the extension, gliding and shear of the materials. Furthermore, the fracture surfaces of the 6- and 2-h treatment appear to retain the same general ductile characteristics with a dimpled appearance. Both indicate good ductility, but the mechanical property data show that the 6-h treatment leads to a slightly higher strength and hardness, and this could correlate with a more refined dimple structure on the fracture surface due to a more uniform distribution of precipitates. In both the 2-h and 6-h treated samples and then aged, the fact that the fracture surfaces still show a largely ductile nature despite the increase in hardness and strength from the treatment process is an indication that the AA2017 alloy maintains a balance between strength and ductility, which is a desirable property in many engineering applications.

### 3.4 Discussion

The extended duration of solution treatment in this study enhanced the dissolution of precipitates, which subsequently allowed for more uniform aging effects, as evidenced by improved mechanical properties. The choice of quenching medium (water or air) significantly influenced the microstructure, as the medium affects the cooling rates. Water quenching, due to its rapid cooling, retained a supersaturated solid solution, which is preferable for achieving finer and more evenly distributed precipitates during aging. This led to higher hardness and tensile strength in comparison to air quenching, which produced coarser precipitates due to slower cooling rates. The age-hardening process significantly affects the alloy's behavior under dynamic loading as well. Tiamiyu et al. [52] emphasize how different temper conditions, specifically T451 and T651, influence the dynamic shock resistance and deformation of AA2017, showing how heat treatment optimizes strength for high strain-rate applications.

The formation of key strengthening phases such as θ-Al_2_Cu and Mg_2_Si was confirmed by SEM/EDS analysis, aligning with theoretical predictions and previous studies. These phases are crucial for the precipitation hardening of the AA2017 alloy. The absence of the S phase is due to the chemical composition of the alloy; in fact, in Al-Cu-Mg alloys with low Cu to Mg ratios (<1), only the α-matrix and θ phase are observed [47]. However, the relatively high content of Si, in association with Mg, which did not participate with Cu in the formation of the S phase, leads to the formation of Mg_2_Si. This study's results emphasized the significant role of these precipitates in enhancing mechanical strength and hardness. The experimental results were corroborated by simulations using JMatPro software, particularly regarding phase transformations and the effects of cooling rates on precipitate formation. The CCT and TTT diagrams generated helped in understanding the critical cooling rates and the transformations that occur at different temperatures and cooling rates.

The higher hardness and tensile strength after the 6-h solution treatment (ST) compared to the 2-h ST can be attributed to the more complete dissolution of solute atoms, leading to a higher degree of supersaturation during quenching. This results in a greater density of finely dispersed Al₂Cu and Mg_2_Si precipitates during artificial aging, which are more effective at pinning dislocations and strengthening the material. In contrast, the 2-h ST may not fully dissolve all the precipitates, leaving behind some coarse particles that contribute less to the overall strength and hardness.

The quenching method also plays a significant role: water quenching (WQ), with its rapid cooling rate, retains a higher level of supersaturation, promoting the formation of finer precipitates during aging. In fact, the mechanical properties of the WQ samples is higher than the AQ samples due to the solid solution strengthening. Air quenching (AQ), being slower, allows some precipitate coarsening during cooling, resulting in larger and fewer precipitates that are less effective in impeding dislocation movement, thus reducing strength and hardness. Different cooling rates during quenching significantly impact corrosion resistance due to microstructural variations. A lower cooling rate leads to the coarsening of grain boundary precipitates and constituent particles, which in turn increases the corrosion rate [53]. Another important aspect to consider is that while high cooling rates are essential for enhancing mechanical properties, they can also lead to significant residual stresses due to the thermal gradients encountered during quenching. Therefore, it is crucial to find a balance between achieving optimal mechanical properties, which require high cooling rates, and minimizing residual thermal stresses by employing lower cooling rates [54].

The mechanical testing results provided clear evidence that optimal heat treatment (500 °C for 6 h, followed by water quenching and aging) leads to the best combination of hardness and tensile strength and effective precipitation of hardening phases. This is confirmed by the fracture surfaces examined after tensile testing, that showed predominantly ductile behavior with features such as micro-void coalescence, which is typical of ductile fracture. This suggests that despite the increase in strength, the material retains adequate toughness. The correct combinations of parameters to obtain a correct precipitation hardening in aluminum alloys. However, further improvements in mechanical properties, such as ultimate tensile strength (UTS) and proof stress, can be achieved through severe plastic deformation techniques like Equal Channel Angular Pressing (ECAP) followed by aging or Accumulative Roll Bonding. However, this process typically results in a reduced elongation at break [[55], [56], [57], [58]]. According to Romero-Resendiz et al. the morphology and size of the Al2Cu and Mg2Si precipitates remained unchanged after the deformation process [59].

These results have direct implications for the production and use of AA2017 in aerospace applications, where both high strength and good fracture toughness are critical. The detailed analysis of precipitates formation and the effects of different heat treatment parameters provide valuable insights for optimizing the processing of this alloy for specific applications.

## 4 Conclusions

This study investigated the effects of solubilization and ageing heat treatment on AA2017 aluminum alloy's microstructure using various techniques, including OM, SEM, and EDS, as well mechanical properties with HV measurements and tensile tests in different orientations. The study's findings lead to the following conclusions:

1. CCT and TTT curves provide phase constitutions for the heat treatment response. Specifically, for this alloy composition, for cooling rates higher than 1 °C/s, there is no precipitation from the supersaturated solid solution (SSSS). This is the case with water quenching (WQ), while in air quenching (AQ), the average cooling rate is not sufficiently rapid.
2. The study concluded that the quenching rate (WQ or AQ) after solution heat treatment and its duration (6h or 2h), affected the precipitation-hardening process, particularly the formation of Al_2_Cu (θ-phase) and Mg_2_Si during aging.
3. The quenching rate (WQ or AQ) after solution heat treatment and its duration (6h or 2h), affected the mechanical properties, with according results on both hardness and tensile tests.
4. The optimal heat treatment for achieving the highest mechanical properties was found to be a solid-solution treatment at 500 °C for 6 h, followed by water quenching and artificial aging at 175 °C for 8 h. This regimen resulted in the highest yield and tensile strengths, as well as hardness, with a slight reduction in ductility compared to the solid-solution treatment at 500 °C for 2 h, followed by water quenching and artificial aging at 175 °C for 8 h, which exhibited a lower proof stress, marginally lower UTS, but slightly higher ductility.

## CRediT authorship contribution statement

**Mauro Carta:** Writing – review & editing, Writing – original draft, Visualization, Validation, Software, Resources, Project administration, Methodology, Investigation, Formal analysis, Data curation, Conceptualization. **Leila Aydi:** Writing – review & editing, Writing – original draft, Visualization, Validation, Software, Resources, Project administration, Methodology, Investigation, Formal analysis, Data curation, Conceptualization. **Pasquale Buonadonna:** Writing – review & editing, Writing – original draft, Visualization, Validation, Supervision, Software, Resources, Project administration, Methodology, Investigation, Formal analysis, Data curation, Conceptualization. **Donato Morea:** Supervision. **Mohamad El Mehtedi:** Writing – review & editing, Writing – original draft, Visualization, Validation, Supervision, Software, Resources, Project administration, Methodology, Investigation, Formal analysis, Data curation, Conceptualization.

## Data availability

Data will be made available on request.

## Ethical approval

Not required.

## Ethics statement

Not applicable.

## Funding

This research did not receive any specific funding.

## Declaration of competing interest

The authors declare the following financial interests/personal relationships which may be considered as potential competing interests: Author Donato Morea is noted as an Advisory Board Member (ABM) of this journal. The other authors declare that they have no known competing financial interests or personal relationships that could have appeared to influence the work reported in this paper.
